# Accounting for repetition and dropout in contemporaneous cross-section learning profiles: Evidence from Rwanda

**DOI:** 10.1016/j.ijedudev.2021.102443

**Published:** 2021-09

**Authors:** Lee Crawfurd

**Affiliations:** Center for Global Development, United Kingdom

**Keywords:** Learning, Assessment, Rwanda, Repetition

## Abstract

How much do children learn in a year of school? Longitudinal data that tracks children over time is scarce in developing countries, and so recent studies estimate learning profiles by comparing the ability of people with different amounts of schooling, at a single point in time. Such estimates of the effect of schooling on learning may be biased upwards by not controlling for repetition and dropout. In this paper I estimate contemporaneous cross-section learning profiles for Rwanda, using data from a nationally representative survey of 3053 children aged six to eighteen. I show how adjusting this learning profile for the total number of years enrolled in school (accounting for repetition and dropout), using detailed schooling histories, reduces the average amount learnt per year by over 60 percent. The learning profile for Rwanda is not just too flat, but flatter than previous estimates suggest.

## Highlights

• Learning profiles allow us to trace learning progress through schooling.
• In this paper I estimate a learning profile for Rwanda using a household-based survey of children.
• I use a detailed schooling history to show that observational learning profiles that don’t explicitly account for repetition and dropout may substantially over-state learning gains per year.

## 1 Introduction

How much do children learn in a year of school? In contrast to many standardised assessments that focus on a single age or grade, learning profiles allow us to trace the relationship between learning achieved and the amount of schooling attained. There are several types of learning profile. A true learning profile, which portrays the amount learned by an individual child or group of children across ages or grades would require panel data that follows the same children and their learning over time. Since panel data is scarce, more efforts have been made to estimate cross-sectional descriptive learning profiles, comparing the learning and schooling of a single cross-section of children or adults. Such cross-sectional descriptive learning profiles however suffer from bias due to ignoring repetition and dropout, which bias upwards the estimated effect of schooling on learning.

In this paper I estimate a cross-sectional descriptive learning profile for Rwanda. I depart from previous estimates for Rwanda that have used data from adults, that are informative primarily about the quality of schools (in terms of learning imparted per year) in the past when adults were in school. Instead I use data from a household-based survey of children. I also make use of a detailed school history record, that allows me to assess directly the magnitude of bias due to repetition and dropout.

Learning profiles in Rwanda have been estimated previously using surveys of adults (Oye et al., 2016; Kaffenberger and Pritchett, 2020). These studies look at the relationship between the highest level of schooling attained by adults, and their probability of being able to read a sentence. Both papers find that learning increases with schooling by more in Rwanda than in most other countries, but also point out that these learning profiles over-state learning gains to the extent that they omit data on dropout and repetition. If we know only the highest level of schooling attained and take no account of repetition, we over-estimate the learning per year of someone who has taken twice as long to reach their highest grade, because they repeated every year. If we take no account of dropout, then our estimates of the effect of schooling are biased upwards by unobserved factors that relate to both learning ability and dropout, such as family income or support. Rwanda has one of the highest rates of dropout across 51 developing countries analysed with relevant Demographic and Health Survey (DHS) data (Pritchett and Sandefur, 2020). This means that the sample of adult women whose highest level of schooling is grade 5 or 6 is more selective in Rwanda than in most other countries.

One approach to dealing with this issue is looking at officially reported average national rates of repetition and drop-out. However adjusting for average national repetition rates does little to change estimates of school quality based on retrospective descriptive learning profiles (Le Nestour and Sandefur, 2020). Instead, data is need on repetition and drop-out of individual children.

In this paper I estimate a contemporaneous descriptive learning profile, using detailed schooling histories for each child to explicitly account for repetition and dropout. I use data of children in several grades taking comparable tests at the same point in time. I show that the size of the over-estimate from a learning profile that does not take this into consideration is substantial – over 60 percent.

The rest of the paper is organized as follows. Section 2 reviews the literature on learning profiles in more detail. Section 3 lays out my research questions. Section 4 describes the study context and presents some basic facts about the Rwandan education system. Section 5 describes the data used. Section 6 presents the methods, and Section 7 the results. I then discuss the results in Section 8, and conclude in Section 9.

## 2 Literature review

Following the typology laid out in Kaffenberger (2019), we can think of four types of learning profiles: causal estimates, a panel learning profile, an adult retrospective learning profile, and a contemporaneous cross-section learning profile.

First, causal estimates of the effect of schooling on learning are scarce due to the rarity of quasi-experimental variation in schooling. Where quasi-experimental studies do exist, they typically focus on a specific grade or level of schooling rather than allowing for estimates of the effect of multiple individual grades. For example Singh (2019) provides causal estimates of the effect of a single year of school on learning, exploiting age cut- offs in enrolment guidelines in a regression discontinuity design. Analysis of a lottery for scholarships in Ghana shows that a year of secondary school increases standardized test scores by 0.1 standard deviations (Duflo et al., 2018).

Second, a panel learning profile is based on longitudinal data following the same students over time, on a vertically-linked assessment. This requires both tracking the same students over time, and an assessment with some identical question items (used for linking). One example is Muralidharan and Zieleniak (2015), who follow a large sample of children as part of a large policy evaluation in Andhra Pradesh, India. Such analysis allows us to measure actual learning progress, and by comparing gains in learning we are able to control for a large amount of unobserved differences between children that might otherwise bias estimates.

Third, adult retrospective learning profiles compare the learning ability of adults who attained different levels of schooling in the past (see for example Beatty et al., 2021; Kaffenberger and Pritchett, 2020). This is useful in telling us something about school quality in countries where other learning assessment data does not exist. By using data on adults, retrospective learning profiles provide estimates of historical rather than current school quality (Le Nestour and Sandefur, 2020). These estimates of school quality also need stronger assumptions about conditional independence based on observed control variables.

Fourth, contemporaneous cross-section learning profiles compare students across grades at a single point in time on a comparable assessment (for example Akmal and Pritchett, 2021; Asadullah and Chaudhury, 2015; Muralidharan et al., 2019; Spaull and Kotze, 2015). Such profiles can provide policymakers with an up to date estimate of the quality of schooling. These observational cross-sectional estimates also require strong assumptions – for example that we are able to control for critical correlates of grade attainment and learning and that there are no unobserved shocks that may affect learning levels in some grades but not others. Other studies have used different approaches to dealing with bias in contemporaneous cross-section learning profiles. First, Le Nestour and Sandefur (2020) control for average repetition rates across countries, showing that this makes little difference to learning profiles. Another approach proposed by Sandefur et al. (2016) is to estimate Lee (2009) bounds on the bias by assuming the maximum possible bias through sorting of different children into different grades (a standard approach to dealing with missing data). This approach suggests that failing to account for dropout may significantly overstate true learning, but the bounds are wide.

## 3 Research questions

In this paper I address three main research questions. First, how steep is the learning profile in Rwanda across grades (or how much do children learn in a year of school)? Second, to what extent do high levels of repetition and dropout bias estimates of learning per year? Third, how do learning profiles vary for different disadvantaged groups – specifically girls, children from low socioeconomic status families, and children with disabilities.

Documenting where learning profiles are flat (there is little learning per year) can highlight issues of over-ambitious learning curricula (Pritchett and Beatty, 2015). Estimating accurate learning profiles for different disadvantaged groups allows us to show the evolution of learning gaps between groups – whether they grow with school or are present at the start of school.

## 4 Context

Rwanda has achieved a reputation for effective service delivery in the years since the genocide against the Tutsis, achieving a rapid increase in primary school enrolment (Akresh and De Walque, 2008; Guariso and Verpoorten, 2018), and better health outcomes than other low-income countries. This success has been due to a political economy in which legitimacy has been based on rapid socio-economic development (Chemouni, 2018), a high-functioning bureaucracy,^1^ and government focus on meeting international development goals (Abbott et al., 2017; Binagwaho et al., 2014).

Despite these successes, learning outcomes remain poor. The World Bank Human Capital project puts learning in Rwanda on an international scale for the first time, by linking Early Grade Reading Assessment (EGRA) comprehension scores from Rwanda to international standardized assessments (Patrinos and Angrist, 2018). On this scale, Rwanda ranked 27th of 41 countries in sub-Saharan Africa for learning in 2018 (compared to 4th and 5th in Africa adult and child survival, respectively). The average child in Rwanda can expect to receive 6.6 years of schooling. Adjusted for the quality of learning, this is equivalent to just 3.8 years in the best performing country on the scale (Kraay, 2018).

A likely explanation for this difference in performance is that government has focused on achieving the Millennium Development Goals, which in education only included enrolment, but in health included *outcomes* (child and maternal survival). Both national and local government targets and performance incentives have focused on the most readily observable aspects of education quality, such as classroom construction rather than teaching and learning outcomes that are more difficult to measure (Williams, 2017). The government has also struggled to ensure adequate supply of basic inputs such as textbooks (Milligan et al., 2017).

Other causes of poor overall educational performance include over-ambitious curricula (Pritchett and Beatty, 2015; van de Kuilen et al., 2019), low teacher pay and support, and the switch of the language of instruction from French to English (which is not yet widely spoken) in 2018. Kinyarwanda remains the language of instruction in grades one to three, but this switches to English from grade four (Williams, 2017).

Rwandan local government is comprised of 416 sectors, 30 districts, and five provinces. Teacher recruitment is managed at the district level. The majority of the population (83 percent) live in rural locations (National Institute of Statistics of Rwanda (NISR) and Ministry of Finance and Economic Planning (MINECOFIN) [Rwanda], 2014). Schooling is primarily public, with just 8 percent of school pupils attending private schools (National Institute of Statistics of Rwanda, 2018).

Rwanda’s official average repetition rate in primary school as reported to UNESCO was 13 percent in 2017, slightly above the average of 10 percent for low- income countries.^2^ Survey-based measures of repetition are much higher. The 2016/17 national household survey estimated a repetition rate of 21 percent in the previous year for those aged 8 and above and attending primary school (National Institute of Statistics of Rwanda, 2018). The Ministry of Education’s Learning Assessment of Rwandan Schools (LARS 3) showed that 80 percent of students in P.6 (grade 6) reported ever having repeated a grade (Burdett and James, 2018). The baseline survey for the USAID Soma Umenye project showed a repetition rate of 27.5 percent in grade one (Allan et al., 2018). Neither of these studies is though able to provide a complete picture of repetition.

Survey-based learning assessments show a clear pattern of poor performance going back several years (Table A1). The Learning Assessment of Rwandan Schools (LARS) published in 2018 showed that only around half of children in the sixth year of primary school (P6) were at the expected level in reading and maths (Burdett and James, 2018). The USAID Fluency Assessment of Rwandan Schools (FARS) found that just 35 percent of P.2 (grade 2) children could read in Kinyarwanda with fluency and comprehension in 2016 (Hebert, 2017).

These learning assessments present much worse results than suggested by retrospective learning profiles based on adults. Oye et al. (2016) show that adult women with five or six years of education are more likely to be literate in Rwanda than in almost all other developing countries for which comparable DHS data exists. The estimated gain in the probability of being literate for each additional grade is higher in Rwanda than for most other countries. This presents a puzzle.

Children are expected to start grade one at the age of seven. Data on preschool provision is mixed. Friedlander et al. (2014) found that 71 per cent of students in their sample reported attending some kind of pre-school care provision, though it was unclear whether this was a formal center with trained caregivers or not. This is substantially higher than the level attending formal pre-primary classes as reported by the Ministry of Education.

## 5 Data

In this paper I use a nationally representative household survey of 8122 school age children (between 6 and 18 years old), conducted between February and April 2017 (Laterite et al., 2017). The survey was conducted by a private survey firm (Laterite) for UNICEF and the Ministry of Education. Data was shared by the Ministry of Education. All children in selected households aged between six and eighteen were assessed. I restrict the sample to include only those tested with Early Grade Reading and Early Grade Mathematics Assessments, enrolled in grade one to six or having dropped out of school, and with data on student and household characteristics, leaving 3053 children from 1788 households. The literacy assessment measures Kinyarwanda (grade 1–3) or English (grade 4) reading comprehension skills, and the numeracy assessment tests basic mathematics skills.

In addition to asking both parents and children for the child’s current grade and whether each child had ever repeated a year of school, enumerators also recorded a full schooling history for each child. This recorded for up to 12 previous years what grade the student was in that year. This schooling history was asked of children, and then checked by enumerators with their parents to ensure agreement. Based on this schooling history, I calculate the number of times that each student repeated a grade, and the total number of years that they were enrolled in any grade between one and six.

Table 1 below presents descriptive statistics. The average age in the sample is 11.5 years, and half are girls. Five percent of children have dropped out and are not currently in school. The mean student has repeated one year of school. Parents report that 18 percent of children have some form of disability, including difficulty seeing, hearing, speaking, with self-care, learing, or making friends. 79 percent live in rural areas. The average number of children’s books at home is 0.3.

**Table 1:** Descriptive Statistics.

Variable	Obs	Age	% Female	Repeated Years	Total Years in School
Out of School	164	15.6	0.52	2.3	7.1
Grade - 1	202	8.4	0.44	0.6	1.7
Grade - 2	662	9.2	0.44	0.6	2.3
Grade - 3	604	10.6	0.46	0.9	3.6
Grade - 4	570	11.9	0.50	1.1	4.9
Grade - 5	521	13.1	0.54	1.3	6.2
Grade - 6	330	14.3	0.53	1.4	7.3
All	3053	11.5	0.49	1.0	4.5

Variable	Has a Disability	Rural	Parent Support Index	Wealth Index	Number of Children’s Books
Out of School	0.18	0.82	−0.50	−0.26	0.34
Grade - 1	0.22	0.72	−0.44	−0.10	0.15
Grade - 2	0.20	0.79	−0.14	−0.10	0.32
Grade - 3	0.19	0.81	−0.00	−0.07	0.34
Grade - 4	0.17	0.76	0.16	0.07	0.34
Grade - 5	0.16	0.80	0.16	0.11	0.36
Grade - 6	0.15	0.78	0.26	0.21	0.47
All	0.18	0.79	0.00	0.00	0.34

I calculate a parent support index based on the extent to which students agreed with a series of seven statements about their parents. Each answer is scored on a five-point Likert scale. This index is standardized to mean zero with a standard deviation of one. The statements are;

(1) Your siblings helped you more with your homework than your parents.
(2) Your parents always knew the solutions to your homework questions.
(3) Your parents think chores and supporting the household business/farm is more important than school.
(4) Your parents forced you to go to school even when you did not want to.
(5) Your parents sometimes asked you to miss a day of school in order to support household business/farm.
(6) Your parents were satisfied with your performance at school.
(7) If there was a problem at home my parents talked to my teachers about it.

Finally, I calculate a standard household wealth measure based on a simple asset index (following Filmer and Pritchett, 2001). This index is the first principal component of detailed list of 25 assets; sofa, chair, bed, table, refrigerator, cooking pots, radio, television, mobile phone, an iron, fan, stove, generator, boat, sewing machine, computer, hand hoe, axe, machete, sickle, bicycle, motorbike, motor vehicle, agricultural plot, and livestock. The index is standardized to mean zero and standard deviation of one.

### 5.1 Assessments

Assessments are based on a sub-set of the Kinyarwanda EGRA and EGMA items used in the REB-USAID Literacy, Language and Learning Initiative (L3) (Hebert, 2017; Laterite et al., 2017). These items were initially developed by the Rwanda Education Board (REB) and EDC, based on international standards for measuring early grade literacy and mathematics, Rwandan national grade-level standards, and the Rwandan competency-based curriculum. A reliability analysis of the mathematics items showed a strong reliability for all four sub-tests (addition, subtraction, multiplication, and division) (Hebert, 2017). Questions were set at four different grade levels covering grades one to four. Questions from each grade level were asked of pupils in that grade and the grade above, meaning that, for instance, grade 1 level questions were asked of both children in grade one and grade two. Grade four questions were asked of students in grade four, five, and six. This overlap in the same question items being asked of students in different grades allows analysis of descriptive learning profiles, showing the increase in learning per grade.

Reading assessments consisted of a simple comprehension task – being given an unlimited amount of time to read a 50 word paragraph, and then answering five questions about this paragraph without referencing the reading passage. The expert group that developed the EGRA assessment deemed that answering four out of five questions correctly was the minimum expectation to be at grade level (Hebert, 2017) (examples are included in Appendix B). A limitation to these assessments is that some of the questions might be guessed without reference to the reading passage. This applies most to the grade four English assessment, but does not affect the validity of the comparison across grades.

In mathematics, grade one children were given nine minutes to answer up to thirty grade one level items. These are split evenly between number discrimination, addition, and subtraction. Grade two children were given either the same 30 grade one items, or 30 grade two items (in addition, subtraction, and multiplication). Grade three children were given either the 30 grade two items, or 40 grade three items (addition, subtraction, multiplication, and division). Grade four children were given either the 40 grade three items, or 40 grade four items. Grade five and six children all received the 40 grade four items. Children were handed a pen and paper and given three minutes to complete each block of 10 mathematics questions.

Data in the released dataset includes the number of items attempted and answered accurately in English and Maths at each question grade level. For mathematics I take the average correct score across all items, and for reading the average correct score. I standardize each outcome to mean zero and standard deviation one.

## 6 Methods

I begin by presenting simple mean outcomes by grade. Next I regress literacy and numeracy outcomes on three different formulations of student grade. First using actual current grade, second using highest grade achieved (thus including those currently out of school), and third the total number of years spent at the relevant grade levels (thus accounting for repetition). I estimate each of the three models with and without student and household control variables. I standardize outcomes to a mean of zero and standard deviation of one. As the data contains multiple children from some households, I cluster standard errors in all models at the household level to account for the non-independence of observations. Finally I estimate heterogeneity in the gradient between learning and grade by different student characteristics.

I also use the learning function (OLS regression of learning on schooling and other covariates) to convert years of schooling into standard deviation effect sizes, following Evans and Yuan (2017) and Baird and Pane (2018).

## 7 Results

### 7.1 Learning profiles in Rwanda

**Fig. 1:**
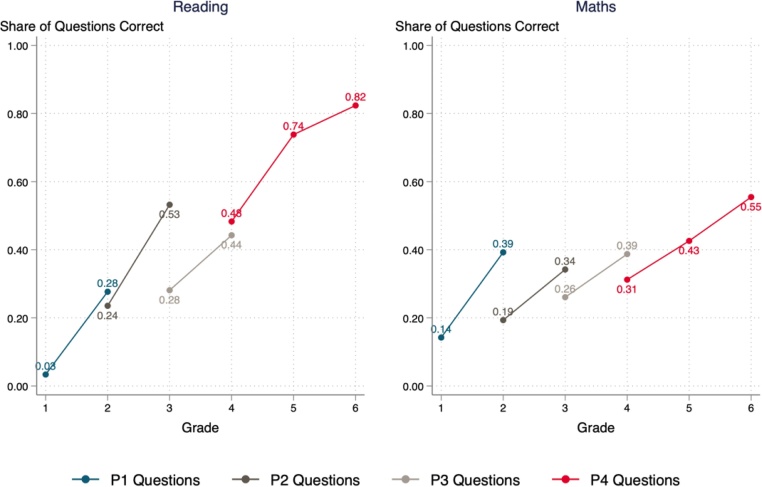
Contemporaneous Cross-Section Learning Profiles for Rwanda.

I begin by addressing the first research question, plotting the learning profile in Rwanda across grades (Fig. 1). Progress does appear to be made between grades. On the grade 1 level assessment, a child in grade 1 answers 14 percent of mathematics questions correctly, while a child in grade 2 answers 39 percent of the same questions correctly. In reading, comprehension level was set by the test designers at being able to answer four out of five questions correctly, something only achieved by grade 6 pupils (on a grade 4 level assessment).

By the end of grade one, children are expected to be able to add numbers between 0 and 99, without carrying a term, where the total sum does not exceed 99. As part of the grade one addition test, children were asked to make the following 10 additions:

2 + 7, 1 + 3, 3 + 2, 4 + 5, 2 + 4,1 + 2, 3 + 4, 7 + 3, 1 + 6, and 6 + 4.

45 percent of children were not able to answer any of these additions correctly in the allocated time. 20 percent did not even attempt to answer any of these questions. On average children were able to compute 28 percent of questions.

### 7.2 Adjusting learning profiles for repetition and dropout

The previous section showed low starting learning levels but a steady improvement across grades. However this picture is biased by ignoring children that have dropped out and repeated multiple grades. In this section I focus on the 1052 children in grades 4, 5, 6, (or out of school) who took the grade four level test. I begin by regressing reading comprehension and mathematics test scores on highest grade attained. This step expands the sample to include out of school children. In this specification I include controls for student and family characteristics, as the objective is to obtain an estimate of school quality. These control variables are student age, sex, family wealth, parental support, living in a rural location, having a disability, and the number of children’s books at home. These controls do not substantially effect the coefficient on highest grade (see Table 2). I control for district level fixed effects in the core specification, but the results are robust to instead controlling for either school or province fixed effects.

**Table 2:** Determinants of Test Scores.

*Mathematics*						
	(1)	(2)	(3)	(4)	(5)	(6)
	In School Only		All		All	
Current Grade	0.623***	0.672***				
	(0.052)	(0.052)				
Highest Grade Achieved			0.627***	0.667***		
			(0.048)	(0.048)		
Years (Grade 4−6)					0.199***	0.251***
					(0.031)	(0.038)
District FE		Yes		Yes		Yes
Controls		Yes		Yes		Yes
Students	933	933	1052	1052	1052	1052
Households	785	785	857	857	857	857
r2	0.147	0.288	0.152	0.295	0.040	0.194

*Reading Comprehension*						
	(1)	(2)	(3)	(4)	(5)	(6)
	In School Only		All		All	
Current Grade	0.392***	0.483***				
	(0.052)	(0.055)				
Highest Grade Achieved			0.377***	0.483***		
			(0.049)	(0.051)		
Years (Grade 4−6)					0.064**	0.159***
					(0.030)	(0.036)
District FE		Yes		Yes		Yes
Controls		Yes		Yes		Yes
Students	933	933	1052	1052	1052	1052
Households	785	785	857	857	857	857
r2	0.061	0.155	0.058	0.153	0.004	0.093

Note: * p < 0.1, ** p < 0.05, *** p < 0.01. The outcome variables for literacy and numeracy are grade four level test scores, standardized to mean zero and standard deviation of one. Controls include age, gender, disabilities, living in an urban area, parental support, family wealth, and number of books in the home. Standard errors are clustered at the household level.

Each grade is associated with an increase in reading comprehension of 0.39 standard deviations, and mathematics of 0.6 standard deviations (Table 2, column 1).

I next replace the highest grade achieved with the total number of years attended school in grade 4 or higher. The coefficient falls by over 60 percent.

**Fig. 2:**
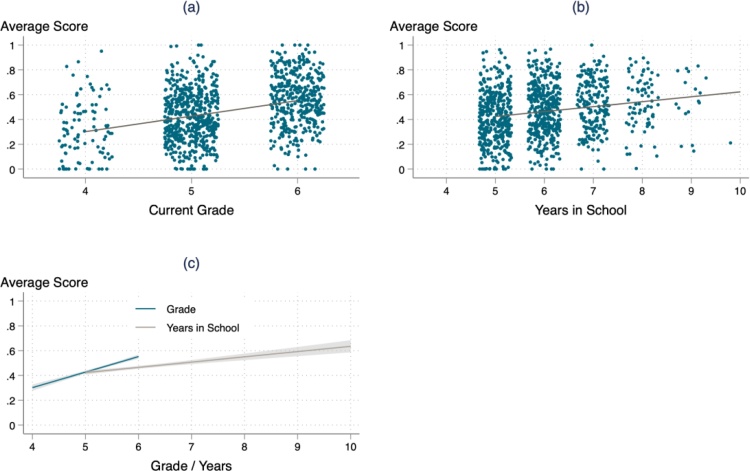
Mathematics Learning Profile Adjusted for Repetition. Note: Fig. 2 (a) shows a scatterplot of learning scores against current grade, with a line of best fit. Fig. 2 (b) shows the same scatterplot against the total number of years that pupils have spent in school, including repeated years.

Fig. 2 again focuses on the three grades for which I have common assessment items – grades 4, 5, and 6. Fig. 2(a) shows the average relationship between learning and highest grade attained, as well as the wide overlap in learning ability within grades. Highest grade attended explains just 6 percent of the total variation in reading comprehension, and 15 percent of the total variation in mathematics. Fig. 2(b) shows the gradient of test scores to total years spent in school in grade 4 or later.

**Fig. 3:**
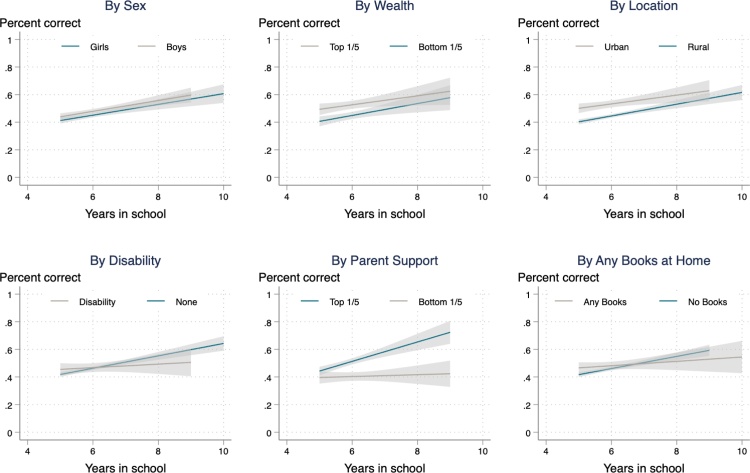
Learning Profiles for Sub-Groups. Note: These figures show average mathematics test scores (the share of questions answered correctly), by years in school, for six subgroups – sex, household wealth, urban or rural residence, disability status, level of parental support, and having books at home. Shaded areas represent 95 per cent confidence intervals.

I then draw learning profiles for five different sub-groups of children to observe whether learning happens at different rates for different students (Fig. 3). Though there are differences in the level of learning, there are little differences in the change in level over time by sex, wealth, urban residence, or having books at home. Disabled children learn less across time than other students, and those with high parental support learn more (see Table A2 for statistical tests).

## 8 Discussion

In this paper, I set out to understand the trajectory of learning in Rwandan primary schools, how naïve estimates are biased by repetition, and how trajectories vary by student characteristics. I find that, contrary to some prior cross-country studies (Oye et al. 2016; Kaffenberger and Pritchett, 2020), learning per year in Rwanda is very low. I also document a large dispersion of ability within classrooms (Fig. 2), making teaching to the full range of abilities incredibly difficult, and may be one reason itself for the poor quantity of learning in each teaching year.

The slope is now much flatter, with pupils achieving grade 4 proficiency only after six or seven years of enrolment. Achieving something closer to mastery (4 of 5 questions correct) takes the average pupil 10 years of being enrolled in school.

I do not find significant differences in the pace of learning for children with different background characteristics. This suggests that although there are inequalities and differences in learning between different sub-groups, these largely exist already at school entry, and do not substantially narrow or widen during school. This data is therefore consistent with the view that learning crises in low income countries are general ones caused by dysfunctional overall systems, rather than driven primarily by inequalities between different groups or individuals (Crouch et al., 2021).

**Fig. A2:**
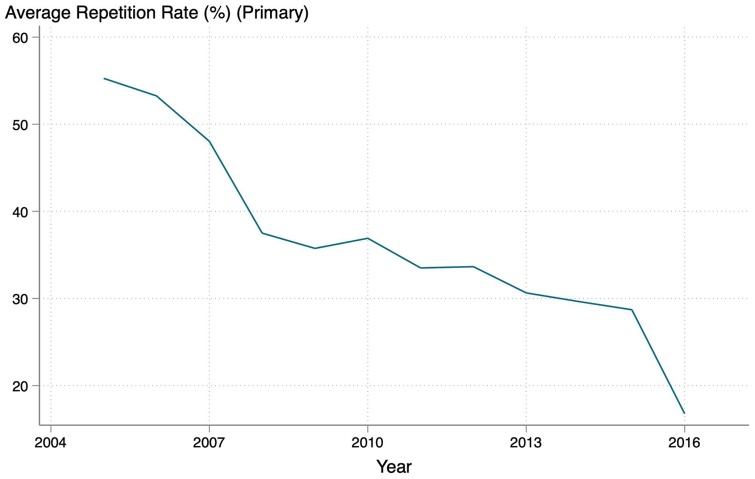
Trend in repetition rate. This figure shows the average repetiion rate by year, calculated as the share of all students in grades 1–6 who were repeating their current grade.

How do we reconcile the large difference between current grade and total years of enrolment, with an official national repetition rate of only 13 percent? Rwanda has in fact seen a large reduction in repetition rates as reported by survey respondents in our sample – from 55 percent in 2005 to 17 percent in 2017. This means that the current repetition rate in our data is close to the officially reported one. Many students in our sample repeated at higher rates in previous years (Fig. A2), and are still captured in this “contemporaneous” data.

### 8.1 Equivalent years of schooling per standard deviation

Considering total time spent enrolled in school, I estimate that each additional year of enrolment is associated with a 0.16 standard deviation improvement in reading and a 0.25 improvement in mathematics (Table 2). These numbers are well within the range of estimates for other developing countries (Bolivia, Colombia, Ghana, Kenya, and Vietnam) (Evans and Yuan, 2019).

This estimate could then be applied to other studies that estimate the effect of interventions to improve learning in Rwanda, in order to provide non-researchers with a more intuitive interpretation of effect sizes than standard deviations. For example a recent impact evaluation of a teacher performance pay scheme in Rwanda reported impacts of 0.09 standard deviations per year on student learning (combining Kinyarwanda, English, Mathematics, Sciences, and Social Studies) (Leaver et al., 2021). If one year in school is associated with 0.16 standard deviations more learning, then a 0.09 standard deviation effect sizes is equivalent to over six months of actual current learning (0.09/0.16). Nsabimana and Mensah (2020) find that a year of school feeding improves maths test scores by 0.18 standard deviations – or 72 percent of business as usual learning. Borzekowski et al. (2019) report effects of watching a children’s educational television show on early learning, with average effects of 0.08 standard deviations, or half a year of learning.

### 8.2 Limitations

This study has several limitations. First, and importantly, the approach relies upon children and parents accurately remembering the grade they were in throughout school, up to twelve years ago.

Second, I am unable to identify children who attend for only part of a year and then repeat the same grade the following year, and thus count these children as having repeated a whole year.

Third, though our learning assessment is more detailed than some single-item assessments used in estimating learning profiles, our assessment is still a simple one, including just five reading comprehension items and 30–40 mathematics exercises per student. Students may learn many more things during a school year that are not captured by this assessment, though it is arguable that reading with comprehension and basic mathematical operations are essential foundational buildings blocks for much other learning.

## 9 Conclusion

In this paper I document the slow pace of learning in Rwanda, using nationally representative household survey data. Students, particularly in early grades, learn little and progress slowly. Using detailed data on student schooling trajectories I estimate the impact of repetition and dropout on estimated learning profiles. Measuring learning against the total number of years enrolled in school rather than just the current or highest attained grade substantially flattens the curve.

As the literacy and numeracy assessments used in this paper are based on Early Grade Assessments that have been used in a range of other countries, there may be scope for drawing comparisons with learning profiles in other countries. Rwanda is an outlier in having a particularly high rate of repetition, but other studies estimating learning profiles using observational data (adult retrospective and contemporaneous cross-section learning profiles) should assess the extent to which estimates might be biased upwards by omitted data on repetition. The direction of this bias is cause for even more pessimism about the effectiveness of school systems in low-income countries in imparting basic skills to all.

An important feature of the learning distribution for policy implications is the marked large bulge of students at the bottom of the distribution who are not learning anything. Significant improvements in average performance could be achieved by ensuring that all children are able to achieve basic foundational literacy and numeracy skills.

The wide variation in student ability within grades poses a serious challenge. The government of Rwanda (and other countries with similar challenges) might look to approaches designed exactly to help teachers deal with such variation in ability (A. Duflo et al., 2020).

## Figures and Tables

**Fig. A1:**
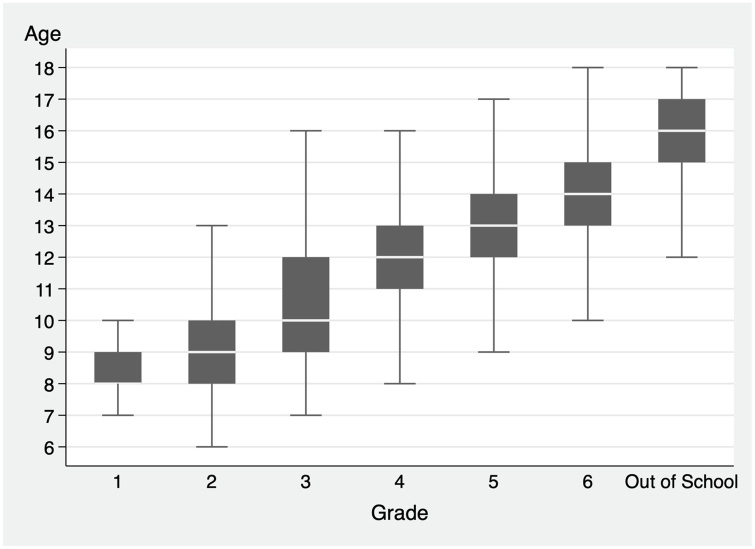
Age Distribution by Grade. Note: This figure shows the wide age distribution of children in each grade. The centre line shows the median age, and the top and bottom of each box show the 75th and 25th percentiles of the distribution, respectively. Lines show the highest and lowest values that are within 1.5 times the inter-quartile range from the box.

**Table A1:** Summary of Rwanda Early Grade Reading Assessments.

Author (Year)	Study	Grades	Report	Fieldwork	Sample	Sample Size	Results	
							Words Per Minute	% at expected level
Allan et al. (2018)	USAID Soma Umenye Baseline	P1	2017/18	2017/18	National	5466 students	P1: 4.5 WPM	
						304 schools		
Burdett and James (2018)	LARS 3	P6 / S3	2018	2017	National	6460 students, 323 schools		P6 - Reading: 57 % - Maths: 59 % (*note that the P6 survival rate is only 50%*)
Outhred and Allen (2017)	LARS 3	P2 / P3	2018	2017	National	7500 Students, 250 schools		P2 - Reading: 60 % - Maths: 34 % P3 - Reading: 28 % - Maths: 15 %
Hebert (2017)	USAID FARS Endline	P1/P2/P3	2017	2016	National	2387 Students 60 schools	P1: 7.7 WPM P2: 24.8 WPM P3: 25.5 WPM P4: 40.1 WPM	P3: 31 %
Moulton (2016)	USAID FARS Midline	P1/P2/P3	2016	2015	National	2580 students	P1: 7.5 WPM P2: 21.5 WPM P3: 25.1 WPM P4: 40.6 WPM	P3: 31 %
						60 schools.		
Moulton (2016)	USAID FARS Baseline	P1/P2/P3	2016	2014	National	1799 students 60 schools	P1: 4.8 WPM P2: 19.2 WPM P3: 22.1 WPM	
	LARS 2	P2 / P5	2014	2014	National	1799 students 60 schools		P2 - Reading: 45 % - Maths: 33 % P5 - Reading 44 % - Maths 38 %
Friedlander et al. (2014)	Literacy Boost	P1	2014/15	2014/15	Gicumbi District	2118 students	P1: 3 WPM	
DeStefano et al. (2012)	USAID EGRA/EGMA	P4 / P6	2012	2011	National	840 students	P4: 26 WPM P6: 48 WPM	
						42 schools		
	LARS 1	P4	2011	2011	National	2420 students		P4: 63 %

Note: The reading fluency standard in Rwanda is 33 words per minute (WPM) (Hebert, 2017). P1 – P6 are the six grades of primary school, and S3 is the third grade of secondary school.

**Table A2:** Interaction Effects.

Mathematics						
	(1)	(2)	(3)	(4)	(5)	(6)
Years	0.195***	0.186**	0.105*	0.198***	0.188**	0.169***
	(0.047)	(0.088)	(0.063)	(0.037)	(0.089)	(0.037)
Female X Years	−0.062					
	(0.061)					
High SES X Years		−0.137				
		(0.109)				
Rural X Years			0.075			
			(0.069)			
Has Disability X Years				−0.216**		
				(0.087)		
Top 1/5 Parent Support X Years					−0.032	
					(0.110)	
Number of Books in HH X Years						−0.019
						(0.025)
Students	1052	1052	1052	1052	1052	
Households	857	857	857	857	857	
R-Squared	0.092	0.096	0.092	0.097	0.094	0.091

Reading Comprehension						
	(1)	(2)	(3)	(4)	(5)	(6)
Years	0.263***	0.263***	0.199***	0.287***	0.109	0.272***
	(0.047)	(0.069)	(0.066)	(0.039)	(0.086)	(0.039)
Female X Years	−0.011					
	(0.057)					
High SES X Years		−0.061				
		(0.093)				
Rural X Years			0.076			
			(0.071)			
Has Disability X Years				−0.183**		
				(0.089)		
Top 1/5 Parent Support X Years					0.264**	
					(0.103)	
Number of Books in HH X Years						−0.042*
						(0.022)
Students	1052	1052	1052	1052	1052	1052
Households	857	857	857	857	857	857
R-Squared	0.194	0.198	0.195	0.198	0.208	0.189

Note: * p < 0.1, ** p < 0.05, *** p < 0.01. High SES is defined as being in the top quintile of the wealth distribution, and high parent support as being in the top quintile of the distribution of a parent support index. Both values are compared to the bottom quintile of the respective indices. Standard errors are clustered at the household level. The sample in all cases is children in grades 4, 5, or 6 or out of school who sat the grade 4 level test. Outcomes are standardized test scores.

## Appendix A Additional Tables and Figures

See Fig. A1

## Appendix B Example Learning Assessment Items

### P1 Kinyarwanda Reading Comprehension (English Translation)

#### Mahoro and Kagabo

Mahoro went to the market to shop but was misled. So he went home and wept. He met Kagabo and asks him to guide him. Kagabo accompanied him to the market. So Mahoro came home very happy.

1 Where was Mahoro going? (To the market)
2 What stopped Mahoro? (He got lost)
3 Who did Mahoro meet when he was lost? (Meets Kagabo)
4 What did Kagabo do for Mahoro? (He accompanied him to the market)
5 How did Mahoro come home? (Excited / very happy)

### P2 Kinyarwanda Reading Comprehension (English Translation)

Kanyange learned to read. Kanyange is in his second year. He loves to read and write. He learned to read letters, words and phrases. He learned to read and write stories. Kanyange takes over clean school supplies and keep them clean. Every day he does not punish his book reading at home. Every evening, he does the homework the teacher gave him.

1 Who is mentioned in the text? (Kanyange)
2 How many years does Kanyange study? (Second year)
3 What does Kanyange like? (Reading / writing)
4 What does Kanyange do every night? (He does the homework the teacher gave him)
5 What does this text teach you? (To love reading and writing)

### P3 Kinyarwanda Reading Comprehension (English Translation)

My name is Mugisha. My country is called Rwanda. Its inhabitants are called Rwandans. I enjoy the talks and lectures on Radio Rwanda that are heard by many. I can't be bothered by these teachings and ridiculous people, who deceive me into pursuing doctrines that oppress me instead of advancing me. As a child, I should always be vigilant, avoiding the temptation of those who want to seduce me. I decided to study well because I love my country. I want to serve the country of my birth.

1 Who are you talking about in this text? (Mugisha)
2 What is his country called? (Rwanda)
3 What makes him happy?
4 (He is interested in the programs broadcast on Radio Rwanda)
5 What is Mugisha's commitment? (Good study)
6 What does this text teach you? (To love (serve) my country)

### P4 English Reading Comprehension

“My name is Kalisa. I like to take care of my body. I drink clean water and eat healthy food. I like to eat fresh fruit and vegetables. It is important to wash your hands before you eat. I like to play games and read books. Sleeping is good for you. It helps your body rest.”

Q1 - Who is talking in the story?

Q2 - What does Kalisa do to take care of his/her body?

Q3 - According to Kalisa, what should you do before eating?

Q4 - What does Kalisa like to do?

Q5 – Why is sleeping good?

#### Primary 1 addition test

Children were asked to make the following 10 additions on paper:

2+7, 1+3, 3+2, 4+5, 2+4, 1+2, 3+4, 7+3, 1+6, 6+4

#### P4/5/6 Multiplication

Children were asked to make the following 10 multiplications on paper:

2x4, 3x3, 5x2, 5x10, 7x3, 4x6, 15x2, 20x10, 6x5, 12 × 10

#### P4/5/6 Division

Children were asked to make the following 10 divisions on paper:

9/3, 4/2, 24/6, 10/2, 15/3, 50/10, 20/5, 55/11, 70/1, 200/2

## Footnotes

1 Rwanda has the best score in the World Bank’s Country Policy and Institutional Assessment for Africa https://www.worldbank.org/en/region/afr/publication/in-five-charts-understanding-the-africa-country-policy-and-institutional-assessment-cpia-report-for-2017

2 World Bank World Development Indicators, accessed 5 October 2020

## References

[bib0005] Abbott P., Sapsford R., Binagwaho A. (2017). Learning from success: how rwanda achieved the millennium development goals for health. World Dev..

[bib0010] Akmal M., Pritchett L. (2021). Learning equity requires more than equality: Learning goals and achievement gaps between the rich and the poor in five developing countries. Int. J. Educ. Dev..

[bib0015] Akresh R., De Walque D. (2008). Armed Conflict and Schooling: Evidence from the 1994 Rwandan Genocide, World Bank Policy Research Working Paper No. 4606.

[bib0020] Allan E.J., Raupp M., Protik A., Ndirangu K., Ramchandani N. (2018). Soma Umenye Impact Evaluation—Year 1 Baseline Report.

[bib0025] Asadullah M.N., Chaudhury N. (2015). The dissonance between schooling and learning: evidence from rural Bangladesh. Comp. Educ. Rev..

[bib0030] Beatty A., Berkhout E., Bima L., Coen T., Pradhan M., Suryadarma D. (2021). Getting children to school but providing little education: comparing Indonesia’s learning profiles in 2000 and 2014. Int. J. Educ. Dev..

[bib0035] Binagwaho A., Farmer P.E., Nsanzimana S., Karema C., Gasana M., Ngirabega J., de D., Ngabo F., Wagner C.M., Nutt C.T., Nyatanyi T., Gatera M., Kayiteshonga Y., Mugeni C., Mugwaneza P., Shema J., Uwaliraye P., Gaju E., Muhimpundu M.A., Dushime T. (2014). Rwanda 20 years on: investing in life. Lancet.

[bib0040] Borzekowski D.L.G., Lando A.L., Olsen S.H., Giffen L. (2019). The impact of an educational media intervention to support children’s early learning in rwanda. Int. J. Early Child..

[bib0045] Burdett N., James Z. (2018). *LARS III*. HEART / Oxford Policy Management..

[bib0050] Chemouni B. (2018). The political path to universal health coverage: power, ideas and community-based health insurance in Rwanda. World Dev..

[bib0055] Crouch L., Rolleston C., Gustafsson M. (2021). Eliminating global learning poverty: The importance of equalities and equity. Int. J. Educ. Dev..

[bib0060] DeStefano J., Ralaingita W., Costello M., Sax A., Frank A. (2012). Early Grade Reading and Mathematics in Rwanda. https://ierc-publicfiles.s3.amazonaws.com/public/resources/Rwanda_EGRA-EGMA-SSME_Final.pdf.

[bib0065] Duflo E., Dupas P., Kremer M. (2018). The Impact of Free Secondary Education: Experimental Evidence From Ghana.

[bib0070] Duflo A., Kiessel J., Lucas A. (2020). External Validity: Four Models of Improving Student Achievement.

[bib0075] Evans D., Yuan F. (2019). Equivalent Years of Schooling: A Metric to Communicate Learning Gains in Concrete Terms. http://documents.worldbank.org/curated/en/123371550594320297/Equivalent-Years-of-Schooling-A-Metric-to-Communicate-Learning-Gains-in-Concrete-Terms.

[bib0080] Filmer D., Pritchett L.H. (2001). Estimating wealth effects without expenditure Data—or tears: an application to educational enrollments in states of india*. Demography.

[bib0085] Friedlander E., Gasana J., Goldenberg C. (2014). Literacy Boost in Rwanda—Reading Assessment Baseline Report.

[bib0090] Guariso A., Verpoorten M. (2018). Armed conflict and schooling in rwanda: digging deeper. Peace Econ. Peace Sci. Public Policy.

[bib0095] Hebert B. (2017). National Fluency and Mathematics Assessment of Rwandan Schools: Endline Report 2016.

[bib0100] Kaffenberger M. (2019). A Typology of Learning Profiles: Tools for Analysing the Dynamics of Learning.

[bib0105] Kaffenberger M., Pritchett L. (2020). Aiming higher: Learning profiles and gender equality in 10 low- and middle-income countries. Int. J. Educ. Dev..

[bib0110] Kraay A.C. (2018). Methodology for a World Bank Human Capital Index. http://documents.worldbank.org/curated/en/300071537907028892/Methodology-for-a-World-Bank-Human-Capital-Index.

[bib0115] Laterite, MINEDUC, UNICEF (2017). Understanding Dropout and Repetition in Rwanda.

[bib0120] Le Nestour A., Sandefur J. (2020). Africa’s Literacy Boom.

[bib0125] Leaver C., Ozier O., Serneels P., Zeitlin A. (2021). Recruitment, Effort, and Retention Effects of Performance Contracts for Civil Servants: Experimental Evidence from Rwandan Primary Schools, American Economic Review.

[bib0130] Lee D.S. (2009). Training, wages, and sample selection: estimating sharp bounds on treatment effects. Rev. Econ. Stud..

[bib0135] Milligan L.O., Tikly L., Williams T., Vianney J.-M., Uworwabayeho A. (2017). Textbook availability and use in Rwandan basic education: a mixed-methods study. Int. J. Educ. Dev..

[bib0140] Moulton J. (2016). Early-Grade Literacy in Rwanda: Taking Stock in 2016. http://idd.edc.org/sites/idd.edc.org/files/Rwanda%20early-grade%20literacy%20sector%20assessment%202016.pdf.

[bib0145] Muralidharan K., Singh A., Ganimian A.J. (2019). Disrupting education? Experimental evidence on technology-aided instruction in India. Am. Econ. Rev..

[bib0150] Muralidharan K., Zieleniak J.Y. (2015). Chasing the Syllabus: Measuring Learning Trajectories in Developing Countries with Longitudinal Data and Item Response Theory.

[bib0155] National Institute of Statistics of Rwanda (Ed.) (2018). The Fifth Integrated Household Living Conditions Survey, EICV5 2016/17: EICV5 Main Indicators Report.

[bib0160] National Institute of Statistics of Rwanda (NISR) & Ministry of Finance and Economic Planning (MINECOFIN) [Rwanda] (2014). Rwanda Fourth Population and Housing Census. Thematic Report: Population Size, Structure and Distribution.

[bib0165] Nsabimana A., Mensah J.T. (2020). Food for Thought: School Feeding and Cognitive Performance in Rwanda.

[bib0170] Outhred R., Allen R. (2017). Learning Achievement in Rwandan Schools—LARS 3 PHASE 1: analysis of Primary 2 and 3 results. HEART Report..

[bib0175] Patrinos H.A., Angrist N. (2018). Global Dataset on Education Quality: A Review and Update (2000-2017).

[bib0180] Pritchett L., Beatty A. (2015). Slow down, you’re going too fast: matching curricula to student skill levels. Int. J. Educ. Dev..

[bib0185] Pritchett L., Sandefur J. (2020). Girls’ schooling and women’s literacy: schooling targets alone won’t reach learning goals. Int. J. Educ. Dev..

[bib0190] Sandefur J., Pritchett L., Beatty A. (2016). learning profiles: the learning crisis Is not (mostly) about enrollment. Society for Research on Educational Effectiveness.

[bib0195] Singh A. (2019). Learning more with every year: school year productivity and international learning divergence. J. Eur. Econ. Assoc..

[bib0200] Spaull N., Kotze J. (2015). Starting behind and staying behind in South Africa: the case of insurmountable learning deficits in mathematics. Int. J. Educ. Dev..

[bib0205] van de Kuilen H.S., Altinyelken H.K., Voogt J.M., Nzabalirwa W. (2019). Policy adoption of learner-centred pedagogy in Rwanda: a case study of its rationale and transfer mechanisms. Int. J. Educ. Dev..

[bib0210] Williams T. (2017). The political economy of primary education: lessons from Rwanda. World Dev..

